# Editorial: Personalized medicine of diabetes retinopathy: from bench to bedside

**DOI:** 10.3389/fendo.2025.1597332

**Published:** 2025-04-01

**Authors:** Kai Jin, Honghua Yu, Wenbin Wei, Andrzej Grzybowski

**Affiliations:** ^1^ Zhejiang University, Eye Center of Second Affiliated Hospital, School of Medicine, Hangzhou, China; ^2^ Zhejiang Provincial Key Laboratory of Ophthalmology, Zhejiang University, Hangzhou, China; ^3^ Zhejiang Provincial Clinical Research Center for Eye Diseases, Zhejiang University, Hangzhou, China; ^4^ Zhejiang Provincial Engineering Institute on Eye Diseases, Zhejiang University, Hangzhou, China; ^5^ Guangdong Eye Institute, Department of Ophthalmology, Guangdong Provincial People’s Hospital, Southern Medical University, Guangzhou, China; ^6^ Beijing Tongren Eye Center, Beijing Tongren Hospital, Capital Medical University, Beijing, China; ^7^ Institute for Research in Ophthalmology, Foundation for Ophthalmology Development, Poznan, Poland; ^8^ Department of Ophthalmology, University of Warmia and Mazury, Olsztyn, Poland

**Keywords:** diabetes retinopathy, etiology, biomarker, diagnosis, treatment

## Introduction

Diabetic retinopathy (DR) stands as a prevalent complication of diabetes mellitus, imposing a substantial burden on individuals worldwide and serving as a leading cause of vision impairment among the middle-aged and elderly populations (1). The progression of DR encompasses various stages, ranging from the initial microvascular alterations to advanced manifestations such as proliferative DR and diabetic macular edema, which significantly compromise visual function (2). Traditionally conceived as primarily a microvascular disorder, emerging evidence underscores the intricate involvement of retinal neurodegeneration in the pathogenesis of DR.

## Pathophysiological insights: beyond microvasculature

DR is a multifaceted disease driven by a persistent hyperglycemic environment characteristic of diabetes. Beyond microvascular alterations, the pathophysiological mechanisms orchestrating DR onset and progression are diverse and interconnected (3). Genetic predisposition, epigenetic modifications, oxidative stress-induced free radical generation, accumulation of advanced glycosylation end products, inflammatory mediators, and dysregulated vascular endothelial growth factor (VEGF) signaling collectively contribute to the intricate landscape of DR pathogenesis (4). This intricate interplay of molecular pathways underscores the heterogeneous nature of DR, posing a significant challenge in devising tailored therapeutic strategies for individual patients.

## Advancements in personalized medicine

The identification of distinct phenotypes of DR, characterized by unique molecular signatures and varying risks for vision-threatening complications, represents a significant breakthrough in patient stratification (5). Molecular profiling techniques, including genomics, proteomics, and metabolomics, have provided valuable insights into the underlying pathophysiological mechanisms of DR (6). By tailoring treatment strategies to match individual patient profiles, personalized medicine holds promise for optimizing therapeutic outcomes and minimizing treatment-related adverse effects.

## Optimization of current treatment strategies

While anti-VEGF therapy and laser photocoagulation remain the mainstays of treatment for diabetic macular edema and proliferative DR, respectively, personalized medicine offers the opportunity to refine and optimize these approaches. Targeted therapies, guided by the specific molecular characteristics of each patient’s disease, have the potential to enhance treatment efficacy and improve long-term visual outcomes (7, 8). By minimizing treatment burden and maximizing therapeutic benefit, personalized approaches aim to improve patient adherence and quality of life.

## Challenges and opportunities

Despite the significant progress made in personalized medicine for DR, several challenges remain. Integration of omics technologies into clinical practice presents logistical and technical hurdles, including standardization of methodologies and interpretation of complex data sets (9). Additionally, the cost-effectiveness and accessibility of personalized approaches must be carefully considered to ensure equitable access to care for all patients (10). However, with continued advancements in technology and collaboration between researchers, clinicians, and industry partners, personalized medicine holds the promise of revolutionizing the management of DR and improving outcomes for patients worldwide.

## Conclusions

In conclusion, the journey towards personalized medicine in DR represents a transformative paradigm shift, poised to revolutionize our approach to patient care and therapeutic decision-making. By embracing the principles of precision medicine and harnessing the power of molecular profiling and biomarker-driven strategies, we can strive towards optimizing visual outcomes and enhancing the quality of life for individuals afflicted with this sight-threatening complication of diabetes.
